# Retrospective diagnosis of a famous historical figure: ontological, epistemic, and ethical considerations

**DOI:** 10.1186/1747-5341-9-10

**Published:** 2014-05-28

**Authors:** Osamu Muramoto

**Affiliations:** 1 Center for Ethics in Health Care, Oregon Health & Science University, 3181 S.W. Sam Jackson Park Rd. UHN-86, Portland, OR 97239-3098, USA

**Keywords:** Retrospective diagnosis, Philosophy of medicine, History of medicine, Medical ethics, Ontology of disease, Epistemology in medicine, Pathography, Socrates, Plato, Frédéric Chopin

## Abstract

The aim of this essay is to elaborate philosophical and ethical underpinnings of posthumous diagnosis of famous historical figures based on literary and artistic products, or commonly called retrospective diagnosis. It discusses ontological and epistemic challenges raised in the humanities and social sciences, and attempts to systematically reply to their criticisms from the viewpoint of clinical medicine, philosophy of medicine, particularly the ontology of disease and the epistemology of diagnosis, and medical ethics. The ontological challenge focuses on the doubt about the persistence of a disease over historical time, whereas the epistemic challenge disputes the inaccessibility of scientific verification of a diagnosis in the past. I argue that the critics are in error in conflating the taxonomy of disease (nosology) and the act of diagnosing a patient. Medical diagnosis is fundamentally a hypothesis-construction and an explanatory device that can be generated under various degrees of uncertainty and limited amount of information. It is not an apodictic judgment (true or false) as the critics presuppose, but a probabilistic (Bayesian) judgment with varying degrees of plausibility under uncertainty. In order to avoid this confusion, I propose that retrospective diagnosis of a historical figure be syndromic without identifying underlying disease, unless there is justifiable reason for such specification. Moreover it should be evaluated not only from the viewpoint of medical science but also in a larger context of the scholarship of the humanities and social sciences by its overall plausibility and consistency. On the other hand, I will endorse their concerns regarding the ethics and professionalism of retrospective diagnosis, and call for the need for situating such a diagnosis in an interdisciplinary scope and the context of the scholarship of the historical figure. I will then enumerate several important caveats for interdisciplinary retrospective diagnosis using an example of the retrospective diagnosis of Socrates for his life-long intermittent neurologic symptoms. Finally, I will situate the present argument in a larger context of the major debate among the historians of medicine and paleopathologists, and discuss the similarities and differences.

## Introduction

Diagnosing medical conditions of a famous historical figure based on evidence found in documents, arts, and other artifacts is a small but popular genre of medical publishing [1]. What is the diagnosis of the illness that tormented Frédéric Chopin for so many years? What neurological disease did Friedrich Nietzsche suffer from for many years before he died? These are typical questions discussed occasionally in medical journals. But this activity is not without criticisms. Some serious scholars in the humanities and social sciences are not happy about this “fun escape of doctors” [1-6]. First, these “hobbyist” historians are not following the methodological disciplines of historiography, literary criticism, and other relevant subject areas of the humanities and social sciences. For example, they often literally interpret the documents in translation without critically analyzing the primary source in the original language. But more importantly, as these retrospective diagnoses become more and more medically sophisticated as medical knowledge advances, these critics are increasingly skeptical about the authenticity of such highly specific and speculative diagnoses, such as alpha-1 antitrypsin deficiency in Chopin [7], which became known only due to the recent advancement in medical technology. These reports often do not consider the possibility that different diseases might have existed in historical time, or the same disease might have been described through different illness experiences that are bound by a particular historical time and place. Hence the critics call them “anachronistic diagnosis” [5,6]. This is an ontological challenge because it questions the existence of a certain disease in historical time. Another challenge is the impossibility of verifying or falsifying the medical hypothesis of retrospective diagnosis for obvious reasons—we cannot examine and test the historical subject. This is an epistemic challenge because it questions the access to historical knowledge. And finally, another important challenge pertains to the ethics and professionalism of those clinicians who render retrospective diagnoses. They publish a diagnosis of a patient with whom they never had a physician-patient relationship, and without any consent. There is such a stark contrast to the diagnosis of their contemporary patients, which is strictly regulated and sanctioned by their professionalism and medical ethics. Even if medical affairs of public figures in historical times are outside the boundaries of medical ethics and professionalism, the question remains what goals such retrospective diagnoses serve. It is often unclear how such a highly specific diagnosis would make any difference in the scholarship of the historical person in question. Without such a discussion these new diagnostic labels are seen by the critics as just a hobby of those physicians who want to exercise their diagnostic acumen for fun. One critic goes so far as to advise journal editors to reject those papers for publication ([3], p144, also see endnote^a^ for a qualification of this criticism). This essay intends to systematically reply to these critics from the viewpoint of clinical medicine, philosophy of medicine, and medical ethics.

Before going into further discussion, however, let me first clarify the concept and terminology of “retrospective diagnosis”. This term has been used at least in four different ways, as shown in the Table 1. First, in clinical medicine, it is a generic term for any diagnosis that is made retrospectively, usually based on new information that was not available in an earlier diagnosis. Postmortem diagnosis based on autopsy is a prototypical retrospective diagnosis. For clinicians, a retrospective diagnosis of a recently deceased patient is routine and uncontroversial, and postmortem diagnosis based on textual evidence might be considered an extension of this practice. In psychiatry, the retrospective diagnosis of mental disorders using phenomenological and psychopathological analysis of written texts has been a branch of study called pathography (Pathographie) [8], which was first introduced by such German neuropsychiatrists as Julius Möbius, Ernst Kretschmer, and Karl Jaspers over a century ago [9]. This methodology is also well established and uncontroversial. But only recently, is the term “pathography” also used in a totally different meaning of illness narratives, particularly in the medical humanities [10]. While the diagnostic pathography has a longer tradition and established usage, the term “retrospective diagnosis” is preferred in recent literature in order to avoid confusion with the illness narrative. Medical diagnosis of historical figures based on textual evidence and other artifacts is the third category of retrospective diagnosis, which is the very subject of this essay. The fourth category of the usage of the term “retrospective diagnosis” is in the field of the history of medicine and paleopathology. The main interest of their investigation is to identify a disease that was experienced and described by the population in a historical time and place, and often diagnosed by medical professionals of that time. While the term “diagnosis” is used, the main endeavor of these medical historians and paleopathologists is the scientific *identification* of a historical *disease*, not so much of diagnosing a *particular individual patient* by analyzing a complex life-long history of illness. The meanings and concepts of all these four categories of retrospective diagnosis are related to each other with many overlaps, but there are also important differences which I will discuss later.

**Table 1:** Different meanings and concepts of “retrospective diagnosis”

**Category of investigation**	**Meaning and concept**
Retrospective Diagnosis in Clinical Medicine	Diagnosis that is made by hindsight, such as after the knowledge of postmortem examination, laboratory tests, and forensic examination
Retrospective Diagnosis in Psychiatry	Pathography, or a psychiatric diagnosis made from written products
Retrospective Diagnosis of a Famous Historical Figure	Diagnosis of a deceased famous historical figure based on biography and other documents and artifacts
Retrospective Diagnosis of Historical Diseases	Scientific identification of historical diseases based on archeological material, documents and artifacts

In what follows, I will first analyze the criticisms of the retrospective diagnosis of famous historical figures in the realms of ontology, epistemology, and ethics/professionalism, and provide my own replies. I will show that the critics’ skepticism of retrospective diagnosis regarding the ontological persistence of disease entities and the epistemic non-verification through diagnostic testing originates in the erroneous conflation of the taxonomy of diseases (nosology) and the act of diagnosing. On the other hand, I will endorse and further develop their criticisms regarding the ethics and professionalism of retrospective diagnosis, particularly the need for clear interdisciplinary scope and goals. Next, I will demonstrate these points using an example of the retrospective diagnosis of Socrates for his life-long intermittent neurologic symptoms. Finally, I will review the relevance of this current proposal with the ongoing discussion among the medical historians and paleopathologists. In this section, I will show important similarities and differences between the retrospective disease *identification* of historical *diseases*, and the retrospective *diagnosis* of a historical *person*.

## Ontological challenges of retrospective diagnosis

While most historians and paleopathologists seem to agree that human anatomy and physiology have not changed or evolved to a significant degree of their interest during the past several thousand years of human history, there has been a tremendous change in human environment, including sanitation, natural climate, and the evolution of other organisms such as microbes and parasites. Leven ([5], p376) introduces the concept of “pathomorphosis” to capture this problem. Based on this concern, he concludes:

The entity tuberculosis itself, like nearly all other disease entities, has achieved the rank of a natural species … What then should prevent us from identifying the present disease entity tuberculosis in descriptions of the past? The intention of this article has been to show that history and its rules should prevent us from such an approach ([5], p383).

Behind this conundrum of identifying the present tuberculosis and the past descriptions of diseases with similar manifestations, there are two kinds of philosophical questions: one is whether Disease X which we recognize as tuberculosis today is the same and identical disease as “phthisis”, “consumption”, or whatever they called in historical time. In this conception, Disease X is one and identical (caused by the same *Mycobacterium tuberculosis* infection) but it is argued that “tuberculosis” and “phthisis” are different because they are conceptualized in a different context with different linguistic, social and cultural meanings. Even if we are dealing with the identical Disease X, biological responses are also different among historical patients due to differences in the immune system and other host factors. Recognition of Disease X by physicians is also different in different historical times due to differences in diagnostic conceptions and technologies. For all these reasons, the critics argue, it is impossible to make a retrospective diagnosis of tuberculosis. This is, however, not a question of the ontology of Disease X because its existence and persistence over historical time is not questioned. What prevents retrospective diagnosis is the differences in human experience, including naming and knowledge, of Disease X. Because this is a form of epistemic argument, I will come back to this question in the next section.

The other way to analyze this conundrum is to consider modern tuberculosis representing Disease X, while historical tuberculosis representing Disease X′. Diseases X and X′ may be related to each other, but they are not identical, or may be clinically similar but may be different entities with different etiology and pathophysiology. If we use the above example, historical phthisis most likely included miscellaneous conditions clinically similar to tuberculosis [11]. Contemporary tuberculosis (Disease X) is caused by *Mycobacterium tuberculosis* whereas the historical tuberculosis (Disease X′) might have included the conditions caused by *Mycobacterium bovis*[12]. In this conception, the underlying diseases themselves are different on top of the historical changes in human experience including the biological responses and classification. This is an ontological question because it questions the persistence of a disease entity itself over time. Many infectious agents are known to evolve in a relatively short period of time (e.g. influenza viruses) and clinical diseases caused by these agents emerge and ebb over time. The ontological and epistemic questions are, however, closely related to each other because, in order to know exactly what Disease X′ was, we still need to know how historical people experienced Disease X′ from historical records. We then face the challenge of the accessibility and verifiability of our historical knowledge of Disease X′. It seems that the epistemic question is pivotal to the ontological questions.

One way to address the persistence of disease entities over historical time is to empirically investigate historical diseases through modern scientific techniques. This is an important area of study in paleopathology. For example, if we can test the remains of past patients, such as bones, teeth, and mummies, we might make a reasonable confirmation that the same disease existed in the past. It seems uncontroversial that the same or closely similar skeletal pathologies and skeletal diseases such as Paget's disease of bone existed in the historical past ([13], pp697-8). Moreover, by extracting ancient DNA from human remains, paleopathologists can provide valuable information regarding the speciation and the identification of the strains of infectious agents. (See Roberts [12] for biomolecular identification of *Mycobacterium tuberculosis* and Haensch et al. [14] for *Yersinia pestis*, the agent for the Black Death, from archeological remains.) While the ontological challenge to retrospective diagnosis may seem to be answered by empirical studies, paleopathologists are still faced with the onerous challenge of epistemology. For example, it is emphasized that paleopathological diagnostic criteria have to derive from modern clinical medicine ([13], pp697-8). Yet paleopathologists themselves are struggling with the challenges of understanding historical diseases within their historical context [15]. In other words, there seems no way to discuss Disease X and X′ other than by analyzing them and describing them in today's methodology and terminology in order to put them into our contemporary discourse. If so, the epistemic problem of historicizing diseases can be a hurdle against any effort to settle the ontological problem. Nevertheless, I agree with the critics to the extent that it is important, whenever we discuss retrospective diagnosis, to address the question whether Disease X and Disease X′ are identical or different, and if different, whether the difference is ontological (difference in disease itself) or epistemic (difference in human experience of the same disease). Often times, we have no definitive answer, but I argue that the absence of evidence for the persistence of a disease is not the evidence for the absence. There is often circumstantial evidence that Disease X and X′ are very close, if not identical, given the approximate continuity of human anatomy, physiology, and chemistry over historical time. We should not dismiss retrospective diagnosis altogether just because we are unable to ascertain that Disease X and X′ are identical, as Leven argues.

A similar but somewhat different concern is expressed by Karenberg [3]. He argues that medical knowledge and nosology change over time, and gives an example of Frédéric Chopin’s diagnoses since 1899. This includes five medical diagnoses related to his respiratory problem, from tuberculosis to cystic fibrosis to alpha-1 antitrypsin deficiency, and five psychiatric diagnoses related to his mental symptoms, from “psychasthenia” to bipolar disorder. Karenberg wrote that these diagnoses represent a “perfect self-referential system” within medicine, and “little appreciated by outsiders, … historians of music or biographers.” “[T]he emerging concepts of disease and medical diagnoses proposed [for Chopin’s case] over the last 120 years strongly correspond with research on these diseases in clinical medicine ([3], pp142-3)”. For historians, it is perplexing that the medical condition that a historical figure had, which is supposed to be fixed as a historical reality forever, changes so frequently in correlation with the progress of modern medicine. “How come Chopin can have such a state-of-the-art modern disease which was not known in his time?” is the typical reaction. Leven also echoes Karenberg by saying that it is “a symptom of an anachronistic self-image of medicine” ([5], p384). As a new conception of disease and diagnosis is introduced, clinicians are tempted to apply such a new label to a historical figure. But to historians, it does not make sense at all to put new labels to a historical event without any new historical evidence.

Behind this challenge is the critics’ doctrine that historical facts in general, and historical diseases in particular, must be interpreted in their historical context, or historicism. Retrospective diagnosis is anachronistic exactly because it tries to diagnose a disease of the past in contemporary terms. This challenge is also complex because there are two prongs. One is whether a historical fact, in this example the fact that Chopin had Disease X, is independent from the historian who describes it, and the other is whether a medical diagnosis, in this example the diagnosis of Disease X in the patient Chopin, *whether it is in contemporary or historical time*, is independent from diagnosticians. It seems that the critics’ challenges are based on the assumption that it is an *observer-independent* fact that a certain disease existed in a historical person whether any historian can know and describe it or any medical professional can diagnose it. That is the historical reality that is independent of observers. Were they to accept *observer-dependent* historical reality, they would have had no qualms about the ever-changing nature of Chopin’s diagnosis over the past 120 years, because the description of his disease and diagnosis would have been all observer-dependent, always analyzed and described from the observer's viewpoint of his time, and necessarily changed over time. We might call their position ontological realism about historical facts in general, and the persistence of diseases and medical diagnoses more specifically. (For the comparison of realism and antirealism about historical facts, see Murphy [16], and about medical diagnosis, see Simon [17]). But antirealists have a ready reply to those realists: how do they *know* that there are observer-independent historical facts and diagnoses? If they assume that Chopin’s disease is one and fixed forever, how do they know and confirm that alleged fact? Once we start analyzing Chopin’s disease using modern methodology and the language of historiography, paleopathology, and modern diagnostics, the result is of course observer-dependent—dependent on us modern historians and diagnosticians—and thus it is completely anachronistic. How could they possibly prove their alleged historical reality in a non-anachronistic way that is independent from our current “anachronistic” methods? Is there an a-historical vantage point (“God’s eye view”) to view diseases of different historical times? As we analyze the critics’ ontological challenges against retrospective diagnosis, it becomes clear that they are intricately entangled with the epistemic challenges, to which I will turn in the next section.

## Epistemic challenges of retrospective diagnosis

Assuming that a historical figure did have a real disease in the past, *how* do we know what disease he had? This is the central question of retrospective diagnosis. There seem to be two competing views. First, one believes not only that a certain disease existed in the reality of a historical person, but also that we should be able to know that reality in a non-anachronistic way if we were provided with an ideal access to that reality. However, we are not able to know it exactly because we have no adequate access to the historical reality. Texts and artifacts are not preserved as medical data, and they should not serve as a means to access the medical reality of the past ([5], p375). The second thought is that one also accepts the existence of the medical reality of the historical figure in the past, but believes that the only thing we can say about this reality is to construct or reconstruct it in our discourse through our own perception using presently available information, particularly texts and artifacts, along with state-of-the-art medical knowledge. While he may believe it is the best approximation of the historical reality, he readily admits that there is no guarantee that such a construction exactly matches the historical reality. Let me call the first argument realist about retrospective diagnosis, and the second antirealist or constructivist about retrospective diagnosis. In what follows, I will reject the former, and defend the latter.

The realists are pessimistic about the possibility of retrospective diagnosis of historical figures. They have to remain agnostic about such a diagnosis. Thus Leven wrote ([5], p373, emphasis added): “Is it possible then to identify these past diseases with our present diseases? This … question cannot be answered with ‘yes’, rather, it *cannot be answered at all* …”. Let me call this argument skeptical agnosticism.

Karenberg also presents a variant of Leven's argument to cast the same doubt about the epistemic access to retrospective diagnosis:

As a final proof (such as a pathoanatomical finding, a lab test or a genetic analysis) usually cannot be provided, it is impossible to falsify or verify a hypothesis of this kind – the “cases” of Alexander the Great, Mozart and van Gogh are excellent examples for this situation. As a matter of fact, a “may-be”-diagnosis concerning a historical patient never can be ascertained in the same way as a modern patient ([3], p142).

Let me call this the verificationist argument, which is a classical doctrine of logical positivism (empiricism) which says that a proposition can be said to be true only if it could conceivably be shown to be false, if false ([18], p60).

What is common between the above skeptical agnosticism and the verificationist argument is the positivist view of medical diagnosis, which takes it as something to be “identified” and “verified”. As Karenberg states, medical diagnosis is equated with laboratory tests and genetic tests and these tests are considered the “final proof”. Since these tests give a positive-or-negative answer most of the time, medical diagnosis also becomes a binary yes-or-no, or true-or-false question. Disease entities are something that you have or you do not have. (The verificationist argument does not necessarily entail a binary judgment, and I am merely asserting that the critics are using these together.) Leven also states ([5], p383), “Disease entities for modern scientific medicine seem to be biological entities.” And this view is not limited to the critics of retrospective diagnosis; most people exposed to current technological medicine perceive medical diagnosis as a binary judgment of having or not having a disease. But I submit that this view is only a contemporary brainchild of modern biomedical sciences.

Diagnostic categories are a man-made construction, and not the product of nature itself or “biological entities” as Leven asserts (See Wilson [19]). As Cunningham [20] states, a disease or a disease concept does not exist independent from the act of diagnosing. He expresses this fact in a simple phrase: “you die of what your doctor says you die of”. A diagnosis that is independent of a diagnostician does not exist even if there may be anatomical, physiological, and chemical changes that are consistent with a disease in a patient. Only after a diagnostician diagnoses a patient by assigning her into a diagnostic category do we say that the disease exists in the patient. Take a simple example of hypertension to illustrate this point. Blood pressure reading is a continuous spectrum from low to high numbers, and the numbers themselves do not represent a disease or normalcy. It is a man-made cut-off point above which is the value for disease or positive and below which is normal or negative that determines the diagnosis of hypertension. Most laboratory tests have a similar man-made cut-off point to differentiate positive or negative. Even pathological and genetic tests have grey zones and human judgment is final to categorize these cases as disease or no disease. But as we become used to these diagnostic categories, we tend to believe that a diagnostic category or nosology itself, such as hypertension or coronary artery disease, is some sort of natural kind that belongs to nature itself independent from human construction. But as any medical practitioner knows, it is a man-made construction which is constantly revised and reconstructed. Good examples of constantly revised diagnostic categories are ICD (International Classification of Diseases) code and Diagnostic and Statistical Manual of Mental Disorders (DSM).

In the premodern era up to the 19th century, diagnoses that patients received were only about symptoms, and there was no concept of differential diagnosis which includes *identifying* disease A against B or no disease, or *verifying* against diagnostic standards. The preoccupation of a medical practitioner was to offer various therapies depending on symptoms [21,22]. In this respect, it is ironical that the critics of retrospective diagnosis who demand historicized diagnoses of a historical figure also demand non-historicist identification and verification in modern terms, which seems to be self-contradictory and anachronistic.

But a more fundamental question is whether an act of medical diagnosis is all about identifying, verifying, and categorizing underlying diseases of the patient. In fact, medical diagnosis has many important aspects, among which identification and verification are only one of these. In the rest of this section, I will enumerate at least five aspects of medical diagnosis that are important and relevant to retrospective diagnosis. First of all, medical diagnosis is a process of hypothesis-making and hypothesis-adjustment. Medical diagnosis is not just a “truth-finding” expedition into the reality of the sick body and mind of the patient, as natural scientists discover a hidden truth of nature. Nor is it just a classification of the patient’s condition into a known taxonomy, as a botanist examines and classifies a newly discovered plant. Medical diagnosis is a dynamic guide for clinical decision-making with constant iteration between hypothesis-making and hypothesis-evaluation and adjustment according to the clinical reality that changes continuously and rapidly [23]. Because the clinician makes decisions under uncertainty, he has to adjust the diagnostic hypothesis on the fly according to what happens next. And this is one of the reasons why the same patient with the same condition can receive many different diagnoses with different focuses and specificity. Each of these is a valid and important diagnosis at each step of medical care, and gives a different level of guidance to the next step.

The second important aspect of clinical diagnosis is that it is fundamentally a probabilistic judgment (something is more likely or less likely) under uncertainty rather than an apodictic judgment (something is true or false) under certainty, as the critics understand. And the iteration of hypothesis-making and hypothesis-adjustment follows Bayesian probabilistic reasoning [18,24]. In the most simplified form, Bayesian reasoning is expressed as: (Posterior Probability) = (Prior Probability) × (Likelihood Ratio), where Posterior Probability is the probability of correct diagnosis after the diagnostic evaluation (hereafter “Post-odds”) and Prior Probability is the probability of correct diagnosis before the diagnostic evaluation (hereafter “Pre-odds”). Likelihood Ratio is the ratio of the true-positive rate divided by the false-positive rate of the evaluation. When the Post-odds increases from the Pre-odds, that means that the probability of the hypothesis being correct increases (or the diagnosis becomes more plausible), and thus the diagnostic evaluation is correct, that is to say the true-positive rate is greater than the false-positive rate. When the Post-odds decreases, the plausibility decreases and the hypothesis is deemed less likely. The clinician must go back to the previous stage to adjust the initial hypothesis or select a different method of evaluation. By iterating this process of hypothesis-making, -evaluation, and -adjustment according to Bayesian reasoning, the diagnosis can achieve a higher probability of certainty.

The third aspect is that the degree of certainty of a diagnosis need not be 100%. There is no such thing as a 100% certain diagnosis, but more importantly clinicians often are satisfied with an uncertain diagnosis depending on the clinical need and context. Such a diagnosis is still “correct” and fully useful in clinical reality. For example, when a physician sees a patient with upper respiratory symptoms and makes a diagnosis of “viral syndrome”, he will not embark on any “truth-finding” expedition to confirm this diagnosis and find out the exact virus that is causing the patient's symptoms. It is a syndromic diagnosis without etiological confirmation. He is merely assigning the patient to a large undifferentiated diagnostic group of miscellaneous viral infections which are self-limited. This is because identification and verification have no use for clinical management, and he knows that the patients with “viral syndrome” recover spontaneously only by symptomatic treatment. On the other hand, if there is an outbreak of a certain viral infection which needs to be contained, he would probably do a test to determine the exact virus, such as SARS or H1N1. This example shows that a clinical diagnosis and a diagnostic category to be employed are dictated by the need and the context of the patient, place, and time. As Edmond Murphy stated, “A major function of diagnosis is precisely to discern where and where not to look in detail”. ([18], p7) It is not that one diagnosis is “true” and the others are “false”.

The fourth aspect of medical diagnosis that is relevant to the current discussion is that the act of diagnosing is a social practice, and medical diagnosis is an explanatory device with many social implications [25]. A clinician is not a natural scientist whose task is to uncover a hidden state of affairs of nature; she is only applying natural sciences to more pragmatic tasks of caring and treating a sick patient, *explaining* the condition, and *prognosticating* the future course of his suffering. When an ancient physician “diagnosed” a patient with epilepsy as “sacred disease”, it was an explanatory device to advise the patient regarding what to do and what to expect [26]. In the same way, when a modern neurologist diagnoses a patient with epilepsy as “mesial temporal lobe epilepsy with hippocampal sclerosis”, it is also an explanatory device to advise the patient for specific treatment and prognosis that are attached to this specific diagnostic category. It is the best *construction* of the state of affairs at the moment of the advancement of medical knowledge but it does not *represent* the entire reality of the patient. That is why medical diagnosis as a construction of concepts has changed and will change any time in the history of medicine [27]. This fact is best seen in syndromic diagnoses of functional disorders (e.g. fibromyalgia, chronic fatigue syndrome) and many psychiatric disorders. Despite the lack of any confirmatory test, these syndromic diagnoses are a highly useful construction of the state of affairs of the patient. Syndrome (meaning “running together” in Greek) is “the aggregate of signs and symptoms associated with any morbid process, and constituting together the *picture of the disease*.” (Steadman’s Medical Dictionary, emphasis added). Syndromes do not have identifiable or verifiable underlying biological entities, yet they are a very useful explanatory device and used more and more. And most importantly for the discussion of retrospective diagnosis, syndromic diagnosis can be made from history alone and is highly successful. In one frequently cited study [28] clinicians could reach the correct diagnosis more than 80% of the time by clinical history alone without examining the patient or obtaining laboratory tests. These diagnoses were mostly syndromic. The remaining 20% of the cases ended up with different diagnoses after examination and/or laboratory tests. But the lesson we learn from this study is that even for those diseases that have known underlying biological entity can still be diagnosed by syndromic diagnosis 80% of the time. And this is a very decent odds for a judgment under uncertainty. If a clinician is provided with the history of the patient's entire life with many witness accounts of symptoms in a form of biography and other historical documents, is it not unreasonable to expect the accuracy of such a syndromic diagnosis even better?

The last but not the least relevant point is that medical diagnosis has several methodological dimensions. One condition can be diagnosed from many different dimensions: by clinical signs and symptoms (clinical diagnosis); by laboratory tests (laboratory diagnosis); by genetic tests (genetic diagnosis); by identifying etiology (etiological diagnosis); by pathological examination (pathological diagnosis). These methodological dimensions are not synonymous labels for the same condition. For example, AIDS (clinical diagnosis) and HIV infection (etiologic diagnosis) are not synonymous; a patient can have the latter without former. Which methodological dimension to be used depends on the clinical context. As already mentioned, the diagnosis of “viral syndrome” (clinical diagnosis) is sufficient without etiological or laboratory diagnosis in certain clinical contexts. In fact, numerous syndromic diagnoses without identifiable underlying biological facts are being used in contemporary clinical medicine. When a clinician makes a retrospective diagnosis of a historical figure based on his works (texts and arts) and the records of behavior (biography), it is impossible to make a laboratory, pathological, or genetic diagnosis since none of these tests are available unless we also have access to some material that can be analyzed by the methods of paleopathology. And since etiological diagnoses almost invariably rely on tests, these are also impossible. That means that retrospective diagnosis makes sense only if it is a clinical diagnosis. For example, the retrospective diagnosis of “alpha-1 antitrypsin deficiency” for Chopin does not make sense because this requires a laboratory test to measure a serum alpha-1 antitrypsin level, which is not available for Chopin. One could still say Chopin had a *clinical syndrome consistent with or similar to* alpha-1 antitrypsin deficiency. Whether such a clinical diagnosis makes sense is debatable, however, given the fact that this condition is clinically very difficult to distinguish from more common and prevalent emphysema or COPD. Because of this inherent methodological limitation of making a retrospective diagnosis beyond clinical and syndromic diagnosis, I suggest that retrospective diagnosis be limited to these methodological dimensions.

To sum up, medical diagnosis has many different aspects and dimensions and need not be “identifying” and “verifying” an underlying biological reality. Skeptical agnosticism and verificationism are based on the erroneous conflation of taxonomizing the imagined natural kinds of diseases with the multifaceted social act of diagnosing a patient. When the critics realize that the taxonomy of diseases against which diagnoses are “identified” and “verified” is nothing more than a dynamic man-made construction of a given historical time, they lose support of their concept of the observer-independent medical and historical reality.

## Retrospective diagnosis as theory construction

So far, I have argued that it is a misguided demand on the part of the critics of retrospective diagnosis to expect any medical diagnosis to be “identifying” and “verifying” a disease in a patient. However, the critics would object to this argument as follows: retrospective diagnosis is not for a patient whom the clinician is seeing in his clinic or hospital. The patient about whom the diagnosis is rendered is a dead historical person whom the clinician never saw. Therefore, many arguments from the logic of clinical diagnosis as discussed in the previous section do not apply to retrospective diagnosis. For example, there is simply no possibility of hypothesis-modification in the diagnostic process of a historical patient, since no new clinical information is available, except for a rare occasion of newly discovered historical documents or artifacts. And above all, the critics would contend that as long as a retrospective diagnosis is presented as evidence for some larger proposition in the humanities and social sciences, it is necessary that such a diagnosis come with acceptable proof or verification, which is unavailable.

I acknowledge that this is indeed a very serious problem, and as long as we treat retrospective diagnosis, or any medical diagnosis for that matter, as an apodictic scientific proposition, it would be very difficult to put it on the table of any scholarly discourse. (This is a common conundrum of epidemiologists who have to rely on the accuracy of clinician's diagnoses for disease identification). Nevertheless, I would like to offer a new perspective to accommodate retrospective diagnosis in a broader scholarly discourse of the humanities and social sciences as well as medicine. As I discussed earlier, modern medical diagnosis pretty much follows Bayesian reasoning under uncertainty. I suggest that we expand this process to a larger scope of retrospective diagnosis. Under the conception of the Bayesian model of probabilistic judgment, unverifiable retrospective diagnosis can be evaluated for its plausibility and coherence in a larger context. This is also nothing new in present-day medical practice; when clinicians do not have any definitive evidence to follow in order to make a clinical decision, they still follow peer reviews and expert opinions. In order to do this, they regularly present difficult cases in clinical conferences, and see if their diagnosis is supported by the peers. Or sometimes, the case is presented to nationally known experts of the disease in question and their agreement can serve as the support. It is an old-fashioned way to “confirm” a diagnosis when there is no evidence to rely on, but it is still one form of Bayesian decision-making because the change of plausibility from Pre-odds to Post-odds by increasing or decreasing the Likelihood Ratio is considered a confirmation or disconfirmation of the diagnosis. I suggest that retrospective diagnosis be evaluated in similar peer-review methods. Currently peer review medical journals seem to do this job in terms of the soundness of diagnostic reasoning. Unfortunately, however, the review does not seem as rigorous as for contemporary case reports, as retrospective diagnoses are viewed as of minor importance, which the harsh criticisms of the critics will only aggravate.

While this peer review process may serve as a process of confirmation about the medical aspect of retrospective diagnosis, it is also important to address the critics’ concern that such medical discussions do not contribute to the scholarship in the humanities and social sciences. In this regard, it is interesting to note that there is a commonality between medical diagnosis and historiographic theories: both are hypotheses that are confirmed or disconfirmed by evidence using probabilistic, not apodictic, judgment. In other words, both medical diagnosis and historiographic reasoning involve the iteration of hypothesis-making and hypothesis-adjustment according to the evidence available. Day and Radick [29] propose two models, the Bayesian and “explationist” (this term is original to Day and Radick) models, to capture this aspect of historiographic reasoning. Let us use a hypothetical case to demonstrate how these models work to confirm or disconfirm hypotheses of retrospective diagnosis and art history.

Suppose a hypothetical legendary painter in his 50’s started painting pictures with the left side of the canvas being less detailed and more abstract. No medical record was available for this historical artist. Art historians thought that this peculiar change in his painting style was due to his artistic maturation and a shift toward more abstract style and technique. Then a clinician presented a hypothesis that this change was due to brain damage in his right hemisphere. (Similar cases are reported by Bäzner and Hennerici [30]). What was the evidence? Suppose the clinician can produce paintings painted by other painters who were known to have right hemispheric brain damage, and their paintings were very similar to the ones painted by the legendary painter in terms of neglecting the details on the left side of the pictures. Or the clinician can cite the diary of the painter's wife who witnessed that the painter somehow did not pay attention to the left side of the tray when he was eating—a telltale sign of left hemineglect, which is a sign of right hemispheric damage. How do we evaluate this new hypothesis based on a retrospective diagnosis? Under the Bayesian model, the question is whether this evidence makes the brain-damage theory more plausible than the style-change theory. Under the “explationist” model, the question is which hypothesis explains the change in his paintings better. The answer comes from further evaluation of the existing and additional evidence that the art historians can produce to support their style-change theory, and further debates will lead to more hypothesis-making and hypothesis-modification.

The point I would like to make from this hypothetical case is that a retrospective diagnosis should be evaluated as a theory which competes with other non-medical theories in a larger context of the humanities and social sciences. As usual with many theories in these fields, it is pointless to expect that such a theory would render an apodictic judgment of true-or-false; most of the time, they are a probabilistic judgment evaluated on a more-or-less plausible or better-or-worse explainable basis, or coherence with other evidence. And in order for such a case report to be incorporated inside a broader context of scholarly discussion, it is important to state what theory and what question a particular case of retrospective diagnosis would address.

## Problems of ethics and professionalism in retrospective diagnosis

It is interesting to note that the act of clinical diagnosis is under the intense scrutiny of medical ethics and professionalism, yet as far as making a diagnosis of a historical figure, there seems to be no such concern. Is there any problem with ethics and professionalism in retrospective diagnosis? In the absence of a clinician-patient relationship, usual principles of clinical ethics do not apply, but that should not be taken as a reason to believe that anything goes. It is important to consider, first of all, why the retrospective diagnosis is made, and what is the impact that it can make on the scholarship and to our society. The recently debated proposal to exhume Chopin’s heart to make a genetic diagnosis of cystic fibrosis is a prime example of ethical concern [31]. Genetic testing of paleopathological material in general carries moral risks. Besides the disputed desecration of the dead, there is a potential impact on the descendents of that person. More generally, there is a concern for publicizing a medical diagnosis without consent from the patient and family.

One could argue that these historical celebrities are immune from privacy protection because their lives are open to the public. Furthermore because they are long dead, the subject of any potential harm no longer exists. Yet, such an argument is still controversial. First, even though philosophers disagree on how a posthumous event, such as damaging a person's reputation by libel and slander, can harm the person when the person to be harmed does not exist, most philosophers are in agreement that posthumous harm does happen [32,33]. Second, even though the record of one's life is open to the public, it is not clear whether the medical information extracted from historical evidence that was not known to the person or family has more weight in the protection of privacy. For example, certain descendants might object to the publication of a diagnosis that potentially tarnishes the reputation of their ancestor. Third, it is also important to consider the integrity of professionalism when publishing potentially private medical information. Even if there is no proven harm to the deceased celebrity and there is no legal violation of privacy, if a clinician publicizes medical information of a celebrity with a paparazzi mentality, some would see a potential of slippery-slope degradation of medical professionalism. Some might ask, “if this clinician is so nonchalant about disclosing the privacy of a dead celebrity, can he do the same thing for me if I die some day?” And lastly, it is also important to remember that some dead celebrities have enthusiastic followers (e.g. certain religious figures) and intense abominators (e.g. certain dictators). It is ethically considerate to take into account the feelings of these contemporary people. For all these reasons, there should be a justifiable scholastic reason that is carefully balanced against potential ethical concerns before initiating such a medical evaluation in the absence of the patient's consent. And such a justification should come from a broader scholarly context and appropriate multidisciplinary peer review. (From this aspect, the rejection by the Polish government to exhume Chopin’s heart seems quite appropriate [34]).

There seem to be at least three scholarly purposes for making a retrospective diagnosis in a historical figure. First it helps understand the influence of a disease on the works (writing, arts, music etc) and behaviors (personal, social, and political conducts) of that person. This is most useful to scholars in the history of art, music, literature, philosophy, religion, and politics, among others. Second, it helps understand through his works and recorded behaviors how it was like to live with the disease in that historical time. This is of interest to medical historians and those who are interested in historical illness narratives. Third, it can also serve as a precious source to learn the life-long history of a particular disease through a medically reconstructed biography. This is of particular interest for clinicians dealing with certain chronic diseases. There may be more than these to justify the publication of a retrospective diagnosis, but the point here is that it is important to clarify how such a diagnosis would contribute to the scholarship of that historical person, or I would call a broader-context question.

In evidence-based clinical medicine, clinicians in general are not advised to do any diagnostic workup unless the result will change the management of the patient. In such cases, certain specifications of the diagnosis are left unknown. Earlier, I mentioned an example of the generic diagnosis of “viral syndrome” in which clinicians do not initiate the tests for the specification of causative viruses unless there is a particular purpose. I suggest that the same principle be applied to retrospective diagnosis. For example, how does the specification of Chopin’s chronic respiratory illness to several different diseases, tuberculosis, COPD, cystic fibrosis, alpha-1 antitrypsin deficiency, make any significant difference in understanding Chopin’s artistic accomplishment, music style, music performance, and Chopin’s biography overall? [3]. In this example, each diagnosis carries a different etiology but the syndromic presentation is similar: all present with chronic intermittent respiratory distress with cough and sputum. In such cases, it is important to clarify how the degree of diagnostic specificity is justified not only by the medical evidence but also by the scholarship of Chopin. Otherwise, a purely technical overspecification of retrospective diagnosis would only alienate non-medical scholars from the entire practice of retrospective diagnosis.

Lastly, is there any role of retrospective diagnosis with little or no scholarly implication? Is it appropriate to speculate a medical diagnosis of a dead celebrity just for fun? Some say it is an “irresistible,” “eccentric pursuit of doctors”, “fun escape for doctors” [1] and medical professionals’ “historical hobby” [4]. Nothing seems to prevent such activities, but I still maintain that the above mentioned caveats be considered when such an account is published. However, I would also argue that there is a problem of advising journal editors and reviewers to reject many of those reports of retrospective diagnosis as critics suggest [1,3]. If journal editors abide by this advice, eventually some important reports of retrospective diagnosis would fall through the cracks and become orphans without a forum for scholarly dialogue. The reason is that the editors of medical journals are not in the position to evaluate the scholarly impact of such reports in the humanities and social sciences, whereas the editors of those non-medical journals can in no way evaluate the authenticity of medical discussions in such papers. As a result, they flatly reject those papers following the critics’ advice, and some important papers are not published in any journals at all. For this reason, it is important that the soundness of the medical diagnosis be evaluated in the peer review process of a medical journal, and at the same time or thereafter, the appropriateness of the historiographical methodology and the scholarly impact of the diagnosis be evaluated by the peer review process of the respective non-medical journals. In this regard, it is also strongly advisable for a clinician to team up with scholars in the respective field from the beginning of the project. By doing so, medical and non-medical peer reviews can be accomplished in parallel.

## Toward a truly interdisciplinary retrospective diagnosis: A case study of Socrates

In this section, I will take stock of what has been discussed so far, and come up with a list of the key elements in retrospective diagnosis that can be taken seriously by scholars of the humanities and social sciences. This list is by no means intended to claim the novelty and originality of this essay, and should be taken as a composite of the current thesis combined with the caveats that have been published by other scholars. I will use a case of Socrates’ peculiar symptoms of hearing a voice and trance-like episodes to demonstrate these points [35]. The original case report was short and did not have all the important elements that I discussed above.

### Hypothesis-construction

The first point to be considered is what question is to be answered by the retrospective diagnosis (“broader-context question”). In order for a retrospective diagnosis to be incorporated in the scholarship of the humanities and social sciences, it is imperative to situate the question in the middle of an important debate. In case of Socrates, there has been an ongoing controversy among ancient philosophers and classicists about the nature of Socrates’ “divine sign”. According to Plato, Socrates had recurrent episodes of hearing a voice ever since childhood that commanded him to stop or refrain from certain actions. Socrates said, “I have a divine or spiritual sign which Meletus has ridiculed in his deposition. This began when I was a child. It is a voice, and whenever it speaks it turns me away from something I am about to do, but it never encourages me to do anything” (*Apology* 31d — all the quotes from Plato hereafter are from [36]). Socrates variously called this voice “divine sign”, “spiritual sign”, “my prophetic power”, or in Greek “*daimonion*”, which visited Socrates unexpectedly and abruptly irrespective of the importance of the context in which he was placed (*Apology* 31d; 40a “[*daimonion*] opposed me, even in small matters”; *Euthydemus* 272e-273a). The duration of this episode was usually very brief, probably a few seconds to a minute at most, and it often came when he was about to initiate an action or speech, though it was quite unpredictable. The voice seemed inarticulate; Socrates never attributed any specific words to the voice. The prevailing theories among ancient scholars include; 1) a *prima facie* interpretation that it was a divine voice and the experience is religious in nature (e.g. McPharran [37], p191), 2) that Socrates referred to his own voice of reason (e.g. Nussbaum [38], p234), and 3) that it was a sort of “hunch” or feeling of monition (e.g. Vlastos [39]). One of the leading scholars of Plato, Gregory Vlastos, was quoted as saying that Socrates’ *daimonion* is “the gravest of the difficulties we all have to face in our effort to make sense of Socrates” ([40], p206), as each theory has its serious flaws, and there has been no consensus. On one hand, some philosopher such as Bertrand Russell [41] raised a question of Socrates being “insane” but this was widely dismissed by other scholars. The attempt to explain Socrates’ behavior outside of his religion and philosophy has been an anathema for ancient philosophers and classicists in general. For example, Mark Joyal [42], referring to such attempts, wrote: “To be sure, it is in research on the divine sign that some of the low points in the history of Socratic scholarship have been plumbed…” Against this backdrop, a clinician can ask: “Is it still possible that Socrates had a certain neurological condition and a retrospective diagnosis can shed a different light on this debate?” Another possible hypothesis might be: “Did Socrates attribute his symptoms of seizures to a god, just as the Hippocratic source informs us that most laypeople those days attributed seizures in general to gods and spirits?” [26].

### Source of information

As the critics repeatedly admonish [3,15,43,44] the textual source has to be as close as possible to the person of interest, and should be interpreted in the original language. This is a basic methodological requirement in the humanities and social sciences, but it is not easy for clinicians to fulfill. It is best achieved by collaborating with scholars of the area of interest. Because Socrates never wrote anything, all surviving records about his remarks and behaviors are from Plato, Xenophon, and Aristophanes, who are all Socrates’ contemporaries and independently left records. The original Greek texts are readily available for investigation, and this project is an interdisciplinary collaboration of a neurologist and a classicist. The relevant point in this retrospective diagnosis is that it is based on the personal observations of intelligent and observant friends of Socrates’. There are several other ancient texts mentioning Socrates, but these were written centuries after his death, and are believed to be less reliable than the records written by his direct contacts. Still, caution is in order when we interpret Plato for the description of Socrates because of Plato’s own bias and admiration toward Socrates.

### Methodology of diagnosis

As discussed in the above Section Epistemic challenges of retrospective diagnosis, the best methodology that yields the most plausible retrospective diagnosis is syndromic; all other methodologies of diagnosis more or less require laboratory tests and are not suitable for retrospective diagnosis of historical figures. If a clinician really wants to specify the diagnosis to the level of pathological, etiological or laboratory diagnosis as opposed to syndromic diagnosis, it is more appropriate and less misleading to add a phrase such as “*a clinical syndrome similar to* or *consistent with*” before the diagnosis. In the case of Socrates, the authors arrived at a syndromic diagnosis of temporal lobe epilepsy based on four independent clinical features ([35], p653): 1) brief, reversible, and episodic neurologic symptoms consisting of involuntary and unexpected hearing of a voice, mostly associated with action (simple partial seizure); 2) intermittent episodes of diminution or arrest of responsiveness and motion with complete recovery except for various degree of memory loss (complex partial seizure); 3) episodes of prolonged unresponsiveness and arrest of motion (complex partial status); 4) childhood onset with relatively benign course into late adulthood. Although *temporal* lobe epilepsy carries in its name a localizing implication of the temporal lobe, temporal lobe epilepsy as a syndromic diagnosis does not imply that the localization of pathology is definitive, and it can be used as a diagnosis without brain imaging studies or electroencephalography. The clinical syndrome of temporal lobe epilepsy can happen as a result of lesions in the frontal lobe or parietal lobe, and this syndromic diagnosis can allow such variations. If, however, the authors specified the diagnosis as “mesial temporal lobe epilepsy with hippocampal sclerosis”, which is the most common type of temporal lobe epilepsy syndrome but requires an imaging study, it would have been a case of overspecification, unless the claim is qualified by the phrase “a clinical syndrome consistent with …” with justifiable reason.

### Ontological consideration

Is there any possibility that the disease being diagnosed did not exist in the particular historical time? As discussed above in the Section Ontological challenges of retrospective diagnosis, this question is fundamentally an empirical question posed to medical historians and paleopathologists, but it is probably very difficult to find an answer. At least it is important to consider the possibility that the disease in question was caused by a new genetic mutation of the host or a mutation of pathogens (such as AIDS according to the current theory) or unique environmental factors (such as nutritional deprivation) at some point of historical time. In the case of Socrates, the difference from most other cases of retrospective diagnosis is that Socrates was not diagnosed as ill or abnormal in his time. Therefore, the retrospective diagnosis does not raise the most common question whether Disease X (temporal lobe epilepsy) and an ancient Disease X′ are the same, but whether Disease X existed in historical time without being recognized as a disease. It is well-known that the generalized forms of epilepsy have existed at least since the time of Hippocrates [26], but the descriptions of non-convulsive epilepsies, such as temporal lobe epilepsy and absence epilepsy, are difficult to come by. However ancient physicians were also remarkably astute in describing non-convulsive auras of epileptics ([26], p37). Since most auras are often a partial seizure, or a small-scale, initial, and non-convulsive phase of larger seizures, and these could stop by themselves without developing into generalized convulsive seizures, it is logical to assume that non-convulsive forms of epilepsy also existed in historical time. In other words, given the fact that generalized convulsive seizures are just the tip of the iceberg of many other convulsive and non-convulsive forms of seizures, the presence of generalized seizure as the “sacred disease” in ancient times is consistent with the coexistence of many other forms of milder and non-convulsive seizures in the background.

### Hypothesis-evaluation

As discussed above in the Sections Epistemic challenges of retrospective diagnosis and Retrospective diagnosis as theory construction, it is important to evaluate the hypothesis on the basis of the Bayesian model or the “explationist” model (more or less plausible or better explainable) rather than whether it is true or false. This can be done in two areas: medical and non-medical criticisms. Hypothesis-evaluation from a medical viewpoint consists mainly in evaluating differential diagnosis. The main competing differential diagnosis for Socrates would be schizophrenia with auditory hallucination. It is important to discuss, for example, the comparison between temporal lobe epilepsy and schizophrenia in terms of the nature and content of the voice that Socrates heard, life-long benign history, age of onset, etc, and which diagnosis can explain these features better. In this case one could argue, for example, that a very brief, non-articulate stereotyped voice is more consistent with auditory seizures than schizophrenic auditory hallucinations; that if Socrates had childhood-onset schizophrenia, it would have been almost impossible for him to maintain his physical and mental health up to age 70, but it would still be possible if he had a mild case of temporal lobe epilepsy. Non-medical evaluation is to compare the retrospective diagnosis with the competing theories presented by scholars in ancient philosophy, classics, and history. For example, the discussion can include which theory explains better the several difficulties of the *prima facie* interpretation of the voice that have already been presented by Socratic scholars, such as the onset in childhood, the occurrence that is unpredictable and unexplainable to Socrates, the vagueness of the voice and message, and the personal nature of “god” who utters the voice. (Socrates never named the source of the voice, and from every aspect it was personal to him. That seems to be one of the reasons that Socrates was charged for “believing in a new god” and executed.) The proponent of retrospective diagnosis might also present a new perspective on the historical Socrates. His bizarre behavior, which is well recognized by ancient scholars as ‘atopia’ (or strangeness), along with his intense religiosity (compared to his contemporaries, not to our current standards), might be better explained by a personality and behavioral disorder associated with temporal lobe epilepsy (this behavioral disorder is often referred to as Geschwind syndrome). Socrates might have been an epileptic savant who was executed because of his eccentric thoughts and behavior that were at least partially attributable to his neurologic disorder.

### Ethical justification

The final element is the ethical consideration. First of all, we need to ask whether making a retrospective diagnosis is justified by its overall goal. We also need to consider any potential harm to the person of interest and his descendents and followers. As discussed above, the retrospective diagnosis of Socrates was prompted by the scholarly debate outside medicine, and it remains to be seen how the Socratic scholars respond. It is also interesting to note from Plato’s record of Socrates’ behavior that Socrates himself was not convinced of the origin of the voice. He was often baffled by unexpected and unexplainable occurrences of the voice. Particularly when he was accused of believing in a new deity and condemned to death, he was clear that he did not create the divine voice, but he was not at all convinced of its origin. It is quite possible that he would have welcomed an alternative explanation of the voice if it had been available. At any rate, there is no conceivable reason that this diagnosis could have harmed Socrates, and if anything it would have benefited him. One can object to such an ethical consideration because it is a moot point for someone who has been dead for more than 2400 years, and historians in general have no qualms about revealing any reality, good or bad or ugly, of a historical figure. However, I still maintain that if it is done particularly by a practicing clinician, it is prudent at least to consider this aspect of retrospective diagnosis for the sake of professional integrity, as discussed above in the Section Problems of ethics and professionalism in retrospective diagnosis.

## The relevance to the ongoing debate among historians and paleopathologists

So far I have focused my discussion on retrospective diagnosis of an individual historical person by means of biographies and other sources for clinical diagnosis. As stated in the Introduction, however, the concept of retrospective diagnosis used for the diagnosis of an individual person from a life-long history has a subtle but significant difference from that used by medical historians and paleopathologists. In this section, I will briefly go over the debate among them and how it relates to the current proposal for a diagnosis of an individual. While this author, who is neither a historian nor paleopathologist, has no qualification to jump into this debate, it is only fair to explain the relevance of this essay to their debate since I have extensively quoted their works and arguments, and incorporated many of them into my discussion.

As already discussed in the Section Ontological challenges of retrospective diagnosis, medical historians and paleopathologists are struggling among themselves with a similar challenge of identifying historical diseases without relying on anachronistic methodology in modern academic discourse. This debate is commonly called among those historians as the “Cunningham debate” [15,20]. On one hand, there are historians who deny the possibility of retrospective diagnosis altogether, such as Wilson [19] and Cunningham [20], because medical diagnosis is essentially a social construction of the time and place in history, which cannot be transferred to a different historical time. On the other hand, there are those who accept the thesis that historical diagnoses are a social construction [43] or “social diagnosis” [44], but do not deny the possibility of retrospective diagnosis altogether, insofar as the investigators take necessary steps to avoid anachronistic pitfalls when interpreting historical sources. They also advocate, as I do in this paper, a team project among medical professionals and historians. Karenberg ( [3] see also endnote^a^), similar to Mitchell [44], also advocates cautious approaches to historical material without ruling out the possibility of retrospective diagnosis altogether, though he does not advocate the team approach.

How does my current approach, then, relate to this debate? As I already stated in the Section Epistemic challenges of retrospective diagnosis, I fully agree with Wilson [19], Cunningham [20], and Arrizabalaga [43] for that matter, that medical diagnoses are fundamentally a social construction, and a disease or a disease concept does not exist independently from the act of diagnosing in a particular historical time and place. In this sense, I respectfully disagree with Mitchell [44], who contrasts between historical diagnosis as “social diagnosis” on one hand, and contemporary diagnosis as “biological diagnosis” on the other. With Cunningham, and against Mitchell, I would submit that every diagnosis, whether historical or contemporary, including those diagnoses using modern bioscientific technologies, which Mitchell is ready to classify as “biological diagnosis”, is still a social construction. It is true that, generally speaking, modern diagnoses rely much more on biology than social factors, but the difference is a matter of degree, not of kinds. But more importantly, no matter how much or how little “biology” is involved in diagnosis, all diagnostic categories are man-made constructions, as I stated in great detail in the section Epistemic challenges of retrospective diagnosis. In other words, all the diagnoses, modern or historical, biological or non-biological, are constructed by physicians who are acting in the midst of their society with all the influences from their social environment, including religion, culture, science, and technologies. (See endnote^b^ for an example). And their diagnoses are constructed to suit the needs of the patient, family, and society by informing, explaining, theorizing, treating, and prognosticating what happened to the sick patient. Identifying disease is only one component of this social practice, and may not be required in many cases. The act of diagnosis always has its social purposes and implications, whether ancient or modern. This is what I meant by saying that all the diagnoses are fundamentally a social practice and social construction, and I suspect that this is what Cunningham meant when he wrote “turning away from *diseases* … and also away from *disease concepts* … and turning it instead toward how *diagnosis happens*” ([20], p16, emphasis original), and “*you die of what your doctor says you die of*” (ibid, p17, emphasis original).

At any rate, Cunningham then argues, correctly I think, that it is impossible to translate a historical diagnosis into *disease identification* in contemporary terms. Where I disagree with Cunningham, and agree with Mitchell and others, is that retrospective diagnosis is still possible. But the reason is totally different from Mitchell, Karenberg and others. I disagree with Cunningham regarding his thesis that retrospective diagnosis is impossible exactly because I fully endorse the other part of his thesis that all medical diagnoses are essentially a social construction. It seems paradoxical, but the key to understanding this claim is my thesis that, as I argued in the Section Epistemic challenges of retrospective diagnosis in great length, a clinical diagnosis as a social construction is an explanatory device and a theory construction that serve many purposes in society. It can serve not only the patient and her family to receive the explanation of what is going on in her, but also serve as an explanation of what happened to a deceased patient. While I agree with Cunningham that we cannot directly transfer a historical disease concept to a contemporary *disease identification*, I am arguing, against Cunningham, that we can still use our contemporary diagnosis as a modern explanatory device and theory construction, to explain what happened to a historical individual in question, just as historians and archeologists are all using state-of-the-art technology and concepts to explain what happened in the historical world. To insist that physicians must use ancient concepts and tools to diagnose an ancient patient is as illogical as to insist that archeologists must use ancient tools and concepts to investigate their archeological sites and materials. (See also [13] p698, “… it is vital that diagnostic criteria have a secure basis which derives ultimately from clinical medicine”. Here I am talking about the tools and concepts of clinical diagnosis and archeological investigation, not the concepts used to interpret historical documents, which of course requires ancient or historicized concepts).

By situating my current thesis in the midst of the Cunningham debate among historians, it seems clear to me that my disagreement with Cunningham derives from the subtle but critically important difference between *retrospective diagnosis as disease identification in historical time*, or the *retrospective construction of historical nosology*, which is the primary concern for historians and paleopathologists such as Cunningham, and *retrospective clinical diagnosis of a deceased individual*, or *retrospective diagnosis as the construction of a medical theory to explain what happened to a historical individual,* which is the primary concern for those clinicians interested in retrospective diagnosis. As I have discussed in great detail, neglecting this subtle but critical difference and conflating disease identification, or nosology, and the social act of clinical diagnosis have resulted in harsh criticism against the retrospective diagnosis of historical individuals. At the same time, in order to avoid this confusion I have proposed in this essay that such a retrospective diagnosis should be limited to *syndromic diagnosis without identifying disease* unless there is justifiable reason for such specification.

## Conclusion

While professional historians and paleopathologists have their own debate and agenda regarding retrospective diagnosis as reviewed in the preceding section, I have focused this essay on only one type of retrospective diagnosis, that of famous historical individuals. Presently, this type of retrospective diagnosis in academic publications receives two polarized evaluations [1]: In one camp, it is dealt with as a hobby of historically minded clinicians who enjoy testing their diagnostic acumen in famous celebrities and uncovering intriguing medical secrets. These reports usually belong to an accessory section for incidental topics or letters to the editor in medical journals. In the other camp, serious scholars of the humanities and social sciences have little interest in those highly specific diagnostic labels generated by clinicians whose methodologies and wild speculations have little justification. Some of these harsh critics that I have argued against are pessimistic about the possibility of retrospective diagnosis altogether, as long as it uses the modern concept of medical diagnosis. One of the aims of this essay has been to narrow the gap between these two camps and try to elevate the status of retrospective diagnosis of famous historical figures in a truly interdisciplinary arena.

But in the end, the distance between me and the critics of retrospective diagnosis turns out to be much narrower than it would seem; we both agree that retrospective diagnosis has to base on historicized interpretation, and the proper methodologies of historiography should be followed. We also agree that more attention should be given to ethics and professionalism of publishing retrospective diagnosis. Where I disagree with the critics is their argument that retrospective diagnosis as historicized knowledge is impossible because the modern scientific verification of historicized diagnosis is simply illogical or akin to oxymoron. I have replied to the critics that their pessimistic skepticism and obsession with the scientific verification of diagnosis is based on their conflation of the act of categorizing diseases in a system of nosology, or disease identification, and the social act of diagnosing a patient from her history, or the construction of a medical theory. Instead of advising journal editors to stop publishing reports of retrospective diagnosis, they should collaborate with those interested medical specialists, exchange ideas and develop a truly interdisciplinary field of retrospective diagnosis. At the same time, I have emphasized the importance of those “hobbyist” clinicians to reach out to non-medical scholars of the relevant field by situating the retrospective diagnosis in a larger theoretical framework instead of toying with their diagnostic acumen. Such a collaboration has already been proposed by some of the cautious proponents of retrospective diagnosis in the history of medicine and paleopathology [43,44]. My hope is that this essay can pave such a collaborative path in the field of retrospective diagnosis of famous historical figures.

## Endnotes

^a^In all fairness, Karenberg [3] does not deny the possibility of retrospective diagnosis altogether; he rejects only those approaches exemplified below, which he calls “naive retrospective diagnosis”. Yet it is unclear how the editors and reviewers of medical journals or any journals outside history and related fields, who are not historians, can identify which report is “naive” and hence to be rejected. They could rather interpret Karenberg’s advice simply as an indication that all the papers dealing with retrospective diagnosis are illegitimate.

^b^As I quoted in the Section Ontological challenges of retrospective diagnosis, Karenberg’s work [3] on the diagnoses proposed for Chopin’s conditions clearly demonstrates that the diagnostic concepts change in parallel with the advancement of modern biomedical science. In other words, contemporary physicians are bound to use diagnostic concepts and tools that are produced by the contemporary culture of modern biotechnology, just as ancient physicians are bound to use diagnostic concepts and tools that are produced by the ancient culture of sciences and religious beliefs in their society. It is not that one diagnosis is social and another is biological, but they are all socially determined. Chopin’s example which Karenberg beautifully demonstrated, even though for a different purpose, clearly attests to this relationship.

## Competing interest

The author has no competing interest.

## Author's information

Osamu Muramoto, MD, MA, DMedSci is senior scholar at the Center for Ethics in Health Care, Oregon Health & Science University, and a board-certified neurologist (retired).
